# Hip fracture incidence and post-fracture mortality in Victoria, Australia: a state-wide cohort study

**DOI:** 10.1007/s11657-023-01254-6

**Published:** 2023-04-29

**Authors:** Miriam T. Y. Leung, Clara Marquina, Justin P. Turner, Jenni Ilomaki, Tim Tran, J. Simon Bell

**Affiliations:** 1 Centre for Medicine Use and Safety, Faculty of Pharmacy and Pharmaceutical Sciences, Monash University (Parkville Campus), 381 Royal Parade, Victoria 3052 Parkville, Melbourne, Australia; 2 Faculty of Pharmacy, University of Montreal, Québec, Canada; 3 Centre de Recherche, Institut Universitaire de Gériatrie de Montréal, Québec, Canada; 4 Faculty of Pharmacy, Laval University, Québec, Canada; 5 Department of Epidemiology and Preventive Medicine, Monash University, Melbourne, Australia; 6 Pharmacy Department, Austin Health, Melbourne, Australia; 7 Faculty of Health Sciences, University of Eastern Finland, Kuopio, Finland

**Keywords:** Hip fracture, Risk factors, Incidence, Mortality, Frailty

## Abstract

***Summary*:**

Hip fractures are a major public health concern. Number of hip fractures cases increased by 20% from 2012 to 2018. Factors associated with post-fracture mortality included men, those who are frail, living in a non-metropolitan region, or residing in a residential aged care facility. Our results are useful for planning healthcare interventions.

**Purpose:**

Hip fractures are a major public health concern in Australia. Data on hip fracture incidence and mortality are needed to plan and evaluate healthcare interventions. The aims of the study were to investigate (1) the time-trend in absolute number and incidence of first hip fractures, and (2) factors associated with mortality following first hip fractures in Victoria, Australia.

**Methods:**

A state-wide cohort study of all patients aged $\ge$ 50 years admitted to a Victorian hospital for first hip fracture between July 2012 and June 2018. Annual age-standardized incidence rates were calculated using population data from Australian Bureau of Statistics. Multivariate negative binomial regression was used to investigate factors associated with post-fracture mortality.

**Results:**

Overall, 31,578 patients had a first hip fracture, of whom two-thirds were women and 47% were $\ge$ 85 years old. Absolute annual numbers of first hip fractures increased by 20%. There was no significant change in age- and sex-adjusted incidence. In total, 8% died within 30 days and 25% within 1 year. Factors associated with 30-day mortality included age (≥ 85 years old versus 50–64 years old, mortality rate ratio [MRR] 8.05, 95% confidence interval [CI] 5.86–11.33), men (MRR 2.11, 95% CI 1.88–2.37), higher Hospital Frailty Risk Scores (high frailty versus no frailty, MRR 3.46, 95% CI 2.66–4.50), admission from a residential aged care facility (RACF) (MRR 2.28, 95% CI 1.85–2.82), and residing in a non-metropolitan region (MRR 1.22, 95% CI 1.09–1.38). The same factors were associated with 1-year mortality.

**Conclusion:**

The absolute increase in hip fractures highlights the need for interventions to reduce fracture risk, especially for those at higher risk of post-fracture mortality, including men and those who are frail, living in a non-metropolitan region, or residing in a RACF.

**Supplementary Information:**

The online version contains supplementary material available at 10.1007/s11657-023-01254-6.

## Introduction

Hip fractures are a major public health concern [1, 2]. Despite decreasing trends in age-standardized rates in most developed countries [3], the global incidence of hip fractures doubled from 2009 to 2019 [4]. The incidence of hip fracture is projected to double in Asia from 2018 to 2050, with an estimated annual cost of USD 15 billion by 2050 [5]. The Australian age-standardized hip fracture rate decreased from 194.6 to 176.1 per 100,000 population between 2006–2007 and 2015–2016 but absolute numbers increased by 18% due to population aging [6, 7]. Incidence of hip fracture is useful as an indication of health system effectiveness and general population health [7].

Hip fractures cost the Australian healthcare system an estimated $1.01 billion in 2017 [8, 9]. The clinical and economic burden of hip fractures can be reduced by targeting post-fracture care to patients at risk of adverse outcomes, e.g., rehabilitation, management of comorbidities, optimizing medications to reduce the risk of falls, and by improving bone health [10]. The clinical outcomes of hip fracture depended on multifaceted risk factors [11]. Frailty, a systemic aging-associated decline in reserve and function [12], is a physiological factor associated with increased mortality; however, few large population-based studies have reported this parameter in association with hip fracture outcomes [13]. Living in a non-metropolitan region has been associated with lower hip fracture incidence but studies have shown mixed associations with mortality [14]. Patients admitted from residential aged care facilities (RACFs) may have increased risk of mortality; however, most studies were single-center studies and had small sample sizes or follow-up periods that were shorter than 1 year [15].

Data on hip fracture incidence and mortality are needed to plan and evaluate healthcare interventions. Victoria is the second most populous state in Australia with 6.5 million inhabitants, over three-quarters of whom live in the greater Melbourne metropolitan area. Our study aimed to investigate (1) the time-trend in absolute number and incidence of first hip fractures, and (2) factors associated with mortality following first hip fractures in Victoria, Australia, between 2012 and 2018.

## Methods

### Data sources

Data were sourced via a data linkage from the Victorian Admitted Episodes Dataset (VAED) and the National Death Index (NDI). The VAED contains admission records from all public and private hospitals in Victoria, Australia. Data was available from 1 July 2006 to 31 June 2018. It contains comprehensive data on each admission, such as information on demographics, diagnoses, admission sources, and discharge destinations. NDI contains dates of all deaths recorded in Registry of Births, Deaths and Marriages across each state and territory of Australia, thus ensuring no or minimal loss to follow-up for mortality. Population statistics by sex and age were extracted from data published by Australian Bureau of Statistics [16].

### Study population

Patients aged 50 years or above and admitted to a public or private hospital for hip fracture (International Statistical Classification of Diseases and Related Health Problems, Tenth Revision, Australian Modification [ICD-10-AM] S72.0-S72.2) [7, 17-19] and discharged between 1 July 2012 and 30 June 2018 were included. A previous Australian study demonstrated the sensitivity of hip fracture ascertainment from routinely collected administrative data was around 95% and the positive predictive value was above 70% [19]. Our study only looked at the first hip fractures and removed any patients with any previous history of hip fractures in the past 6 years. This minimized the chance of having false positives which mostly came from inaccurately identifying subsequent episodes as new hip fractures. The study population was limited to 50 years or older because younger patients would have been likely to have sustained traumatic or pathologic hip fractures. All primary and secondary diagnoses, defined as medical conditions arising at or during hospitalization, were used for index hip fracture identification, while associated diagnoses defined as conditions not arisen nor treated during hospitalization were excluded [20]. Patients with hip fractures in the 6 years prior to index hip fracture between 1 July 2012 and 30 June 2018 were excluded.

### Incidence

Only index hip fracture was included in calculation for first incident hip fractures. Any subsequent hip fractures were excluded from analyses. For patient characteristics of our cohort, data on demographics, pre-existing comorbidities, frailty status, and type and region of residence were extracted. Demographics data available included age and sex. Pre-existing comorbidities were identified using all ICD-10-AM diagnoses recorded up to 6 years prior to index hip fracture date, excluding diagnoses recorded at index hospitalization (Supplemental Table 1). Quan’s Charlson Comorbidity Index (CCI) was calculated using ICD-10-AM diagnosis codes [21, 22]. Frailty was assessed using the validated Hospital Frailty Risk Score (HFRS) calculated from ICD-10-AM diagnosis codes recorded within 2 years prior to admission for index hip fracture. This is to reflect the frailty status at baseline [23]. HFRS was categorized to no frailty risk (HFRS = 0), low frailty risk (HFRS > 0 and < 5), intermediate frailty risk (HFRS 5–15), and high frailty risk (HFRS > 15) for analysis. Data on admission from and discharge to residential aged care facilities (RACFs) and region of residence were extracted from hospitalization record. Region of residence was derived from statistical local area of residence recorded. All areas classified under the Department of Health Human Services Region as Eastern metropolitan, Southern metropolitan, or North-western metropolitan were grouped as metropolitan; all the others were grouped as non-metropolitan [24]. To account for transfers between hospitals, rehospitalizations within 1 day were combined with the original hospitalization and considered as a single continuous hospitalization, to maximise capturing information on RACF and region of residence across index admission .

### Mortality

Only patients who were discharged before 1 July 2017 were included in mortality analysis to ensure complete follow-up period of 1 year to avoid bias from time-varying mortality. Thirty-day mortality and 1-year mortality were defined as death within 30 days or 1 year after the date of first hip fracture admission. The follow-up period was limited to 1 year to avoid possible bias if patients had different lengths of follow-up. Time at risk for mortality was defined as time to date of death or end of follow-up, whichever earlier. Dates of death from NDI were used to ascertain mortality. For patients who died during their hospital admission, the date of death was defined as the date of discharge in VAED or the date of death in NDI, whichever occurred first. Risks of mortality associated with age, sex, pre-admission HFRS, type, and region of residence were investigated. Individual pre-existing comorbidities and CCI were excluded from the model because HFRS is a composite score of 109 diagnoses codes and additional inclusion may introduce collinearity. Sensitivity analysis was conducted using ICD-10-AM diagnoses recorded 2 years prior to discharge date (i.e., including diagnoses recorded during index hospitalization) to calculate HFRS.

### Statistical analysis

The annual incidence of first hip fractures was calculated as the number of patients discharged following hip fracture during each financial year (i.e., 1 July to 30 June) divided by the Victorian population that year [25]. Victorian population by sex and 1-year age strata at baseline for each financial year were extracted from census data published by Australian Bureau of Statistics [16]. Age-standardized incidence rates were calculated based on 5-year age categories as per the data available for our cohort, using the Victorian population in 2001 as the standard population per recommendation of the Australian Bureau of Statistics [26]. The incidence of death per 100 person-year was also calculated. To assess the trends in incidence and mortality, the year of the admission was included as an independent variable in the negative binomial regression model with age and sex included for adjustment. Risk factors of mortality were analysed by estimating the adjusted mortality rate ratios (MRRs) and their 95% confidence intervals (CIs) using negative binomial regression. The model was adjusted for year of first hip fracture, sex, age, HFRS, type, and region of residence to estimate the independent effects of the variables. Negative binomial regression was used as overdispersion of the data rendered Poisson regression inappropriate for incidence and violation of proportional hazards assumption rendered Cox regression inappropriate for survival analysis [27]. Subgroup analyses stratified by sex were conducted to examine sex differences. All analyses were performed using R (version 4.0.0) and SAS (version 9.4).

### Ethics

The study was approved by the Australian Institute of Health and Welfare Ethics Committee (EO2018-4–468) and Monash University Human Research Ethics Committee (14,339).

## Results

Overall, 31,578 patients were admitted and discharged following hip fractures from July 2012 to June 2018 (Table 1). Two-thirds (*n* = 21,813) of patients were women and 46% (*n* = 14,792) were aged 85 years and over. One-quarter of the patients (*n* = 8537) had multimorbidity (CCI $\ge$ 3). One-quarter (*n* = 8,328) had low pre-admission frailty risk (HFRS > 0 and < 5), another quarter (*n* = 7,943) had intermediate pre-admission frailty risk (HFRS 5–15), and 4% (*n* = 1164) had high pre-admission frailty risk (HFRS > 15). One-third (*n* = 10,067) of patients resided in a non-metropolitan region pre-admission. Around 5% (*n* = 1531) of patients were admitted from a RACF and 18% (*n* = 5513) of patients were discharged to a RACF.

**Table 1:** Baseline characteristics of patients with incident hip fractures from 2012–2013 to 2017–2018 by sex

	Overall (*n* = 31,578)	Women (*n* = 21,813)	Men (*n* = 9765)
Age, *n* (%)			
50–65	2364 (7.5)	1333 (6.1)	1031 (10.6)
65–74	4304 (13.6)	2779 (12.7)	1525 (15.6)
75–84	10,118 (32.0)	6955 (31.9)	3163 (32.4)
≥85	14,792 (46.8)	10,746 (49.3)	4046 (41.4)
Year, *n* (%)			
2012–2013	4676 (14.8)	3254 (14.9)	1422 (14.6)
2013–2014	5039 (16.0)	3501 (16.1)	1538 (15.8)
2014–2015	5452 (17.3)	3841 (17.6)	1611 (16.5)
2015–2016	5338 (16.9)	3725 (17.1)	1613 (16.5)
2016–2017	5427 (17.2)	3731 (17.1)	1696 (17.4)
2017–2018	5646 (17.9)	3761 (17.2)	1885 (19.3)
Admission source, *n* (%)			
RACF	1531 (4.8)	1070 (4.9)	461 (4.7)
Home-dwelling and other^a^	30,047 (95.2)	20,743 (95.1)	9304 (95.3)
Discharge destination, *n* (%)			
RACF	5513 (17.5)	3949 (18.1)	1564 (16.0)
Home-dwelling and other^a^	26,065 (82.5)	17,864 (81.9)	8201 (84.0)
HFRS, *n* (%)^b^			
0	14,143 (44.8)	10,263 (47.0)	3880 (39.7)
> 0 and < 5	8328 (26.4)	5706 (26.2)	2622 (26.9)
5–15	7943 (25.2)	5179 (23.7)	2764 (28.3)
> 15	1164 (3.7)	665 (3.0)	499 (5.1)
CCI, *n* (%)^c^			
0	14,877 (47.1)	10,998 (50.4)	3879 (39.7)
1–2	8164 (25.9)	5761 (26.4)	2403 (24.6)
$\ge$ 3	8537 (27.0)	5054 (23.2)	3483 (35.7)
Region of residence, *n* (%)^d^			
Metropolitan	21,511 (68.1)	14,896 (68.3)	6615 (67.7)
Non-metropolitan	10,067 (31.9)	6917 (31.7)	3150 (32.3)
Comorbidities^b^			
Cardiovascular and cerebrovascular diseases	15,853 (50.2)	10,392 (47.6)	5461 (55.9)
Chronic kidney disease	6793 (21.5)	4051 (18.6)	2742 (28.1)
Dementia	1916 (6.1)	1341 (6.1)	575 (5.9)
Diabetes mellitus	5476 (17.3)	3458 (15.9)	2018 (20.7)
Previous falls	9207 (29.2)	6512 (29.9)	2695 (27.6)
Osteoporosis	7592 (24.0)	4697 (21.5)	2895 (29.6)

Abbreviations: *ATC*, Anatomical Therapeutic Chemical classification defined by World Health Organization; *CCI*, Charlson Comorbidity Index; *HFRS*, Hospital Frailty Risk Score; *RACF*, residential aged care facilities

^a^Including admission from private residences, transition care program, mental health accommodation, and transfers from other healthcare organizations

^b^Weighted score calculated using diagnoses within 2 years prior to admission. ^c^Based on diagnoses within 6 years prior to admission

^d^Based on Department of Health Human Services Region classification

### Incidence

The absolute hip fracture numbers increased by 20% from 2012–2013 (*n* = 4676) to 2017–2018 (*n* = 5646). However, the age-standardized incidence rates remained relatively stable across the years with less than 5% increase from 2012–2013 to 2017–2018 (Table 2). After adjusting for age and sex, the increase in incidence was not statistically significant (Table 2). The relative risk of a first hip fracture was approximately 50-fold in the oldest age group ($\ge$ 85 years) compared with the youngest (50–64 years) in both sexes, while it was 1.6-fold in women compared with men after adjustment (Supplemental Table 2). Results remained largely similar in sex-stratified analysis, except for slight but statistically significantly higher incidences in women from 2013–2014 to 2015–2016 and 2017–2018 in men when compared to the first year (2012–2013) (Supplemental Table 2).

**Table 2:** The absolute numbers, age-standardized incidence rate, and the age- and sex-adjusted incidence rate ratios (IRR) of first incidence from 2012–2013 to 2017–2018

Year	Number	Crude incidence rate (per 100,000 population)	Age-standardized incidence rate (per 100,000 population)	IRR for incidence [95% CI]^a^
2012–2013	4676	82.7	69.9	1.00 (reference)
2013–2014	5039	87.3	73.1	1.03 [0.95–1.12]
2014–2015	5452	92.5	76.7	1.07 [0.99–1.16]
2015–2016	5338	88.6	72.9	1.03 [0.95–1.12]
2016–2017	5427	87.9	72.2	1.02 [0.94–1.11]
2017–2018	5646	89.3	73.3	1.08 [1.00–1.17]

Abbreviations: *IRR*, incidence rate ratio; *CI*, confidence interval

^a^Adjusted for age and sex

### Mortality

In total, 2180 (8%) and 6373 (25%) patients died within 30 days and 1 year after hip-fracture admission, respectively. Among those who died within 30 days and 1 year, 1500 (69%) and 1930 (30%) died during their hospital admission, respectively. The mortality rate did not change significantly from 2012–2013 to 2017–2018. The age- and sex-adjusted MRR of 30-day and 1-year mortality rates across the years when compared to 2012–2013 did not show any significant difference (Table 3). Results remained similar in sex-stratified analysis (Supplemental Table 3).

**Table 3:** The absolute numbers, mortality rate (MR) per 100 person-year, and the age- and sex-adjusted mortality rate ratios (MRR) of 30-day mortality and 1-year mortality from 2012–2013 to 2016–2017

Year	Deaths in 30 days	Deaths in 1 year	Number of patients followed up	MR for 30-day mortality	MR for 1-year mortality	MRR for 30-day mortality [95% CI]^a^	MRR for 1-year mortality [95% CI]^a^
2012–2013	386	1139	4676	105	29.5	1.00 (reference)	1.00 (reference)
2013–2014	427	1247	5039	109	30.0	1.04 [0.87–1.25]	1.00 [0.84–1.18]
2014–2015	467	1326	5452	110	29.5	1.04 [0.88–1.25]	1.02 [0.86–1.20]
2015–2016	457	1331	5338	110	30.3	1.05 [0.88–1.25]	1.05 [0.89–1.24]
2016–2017	443	1330	5427	104	29.7	0.97 [0.81–1.16]	0.97 [0.82–1.14]

Abbreviations: *MR*, mortality rate per 100 person-year; *MRR*, mortality rate ratio; *CI*, confidence interval

^a^Adjusted for age and sex

Men had double the risk of mortality within 30 days (MRR 2.11 [95% CI 1.88–2.37]) and 1 year (MRR 2.60 [95% CI 2.34–2.89]) than women (Table 4). Increasing age was associated with higher risk of 30-day (≥ 85 years old versus 50–64 years old, MRR 8.05 [95% CI 5.86–11.33]) and 1-year mortality (≥ 85 years old versus 50–64 years old, MRR 17.85 [95% CI 14.29–22.37]) (Table 4).

**Table 4:** Mortality rate ratios (MRRs) of risk factors for 30-day mortality and 1-year mortality based on multivariate negative binomial regression

Parameter	30-day mortality MRR [95% CI]^a^	1-year mortality MRR [95% CI]^a^
Year		
2012–2013	1.00 (reference)	1.00 (reference)
2013–2014	1.05 [0.88**–**1.26]	1.04 [0.88**–**1.22]
2014–2015	1.06 [0.89**–**1.26]	1.02 [0.87**–**1.20]
2015–2016	1.02 [0.85**–**1.22]	1.03 [0.88**–**1.21]
2016–2017	0.97 [0.82**–**1.16]	0.98 [0.84**–**1.15]
Sex		
Women	1.00 (reference)	1.00 (reference)
Men	2.11 [1.88**–**2.37]	2.60 [2.34**–**2.89]
Age		
30–64	1.00 (reference)	1.00 (reference)
65–74	1.39 [0.95**–**2.05]	1.86 [1.44**–**2.41]
75–84	3.52 [2.55**–**4.99]	5.35 [4.26**–**6.74]
≥85	8.05 [5.86**–**11.33]	17.85 [14.29**–**22.37]
Hospital Frailty Risk Score^b^		
0	1.00 (reference)	1.00 (reference)
> 0 and < 5	1.38 [1.20**–**1.59]	1.91 [1.69**–**2.15]
5–15	2.32 [2.03**–**2.66]	4.30 [3.81**–**4.85]
> 15	3.46 [2.66**–**4.50]	7.95 [6.21**–**10.22]
Type of residence		
Home-dwelling and other^c^	1.00 (reference)	1.00 (reference)
Residential aged care facilities	2.28 [1.85**–**2.81]	3.56 [2.91**–**4.37]
Region of residence^d^		
Metropolitan	1.00 (reference)	1.00 (reference)
Non-metropolitan	1.22 [1.09**–**1.38]	1.36 [1.22**–**1.51]

Abbreviations: *MRR*, mortality rate ratio; *CI*, confidence interval

^a^Adjusted for year, sex, age, HFRS, type, and region of residence

^b^Weighted score calculated using diagnoses within 2 years prior to admission

^c^Including admission from private residences, transition care program, mental health accommodation, and transfers from other healthcare organizations

^d^Based on Department of Health Human Services Region classification

Patients with higher pre-admission frailty risk had increased risks of mortality after hip fractures. At 30 days after hip fracture, patients with high frailty risk had higher risk of mortality (MRR 3.46 [95% CI 2.66–4.50]) compared to those with no frailty risk. Higher risk of 30-day mortality was also found in patients with intermediate (MRR 2.32 [95% CI 2.03–2.66]) and low frailty risk (MRR 1.38 [95% CI 1.20–1.59]) (Table 4). The increase in risk of 1-year mortality was more marked than that associated with 30-day mortality at each level of frailty risk, with high frailty risk at MRR 7.95 (95% CI 6.21–10.22), intermediate frailty risk at MRR 4.30 (95% CI 3.81–4.85), and low frailty risk at MRR 1.91 (95% CI 1.69–2.15) (Table 4). Sensitivity analysis using diagnoses within 2 years prior to discharge date to define frailty showed similar results (Supplemental Table 4).

The region and type of residence were also associated with mortality. Patients admitted from a RACF had greater risk of 30-day mortality (MRR 2.28 [95% CI 1.85–2.81]) and 1-year mortality (MRR 3.56 [95% CI 2.91–4.37]) compared to patients not admitted from home-dwelling and other settings, while residence in a non-metropolitan region, compared to residence in a metropolitan region, had MRRs of 1.22 (95% CI 1.09–1.38) and 1.36 (95% CI 1.22–1.51) for 30-day mortality and 1-year mortality respectively (Table 4). Results remained similar in sex-stratified analysis (Supplemental Tables 5 and 6).

## Discussion

Our study highlights a 20% increase in the absolute annual number of incident hip fractures from 2012 to 2018, despite the age- and sex-standardized incidence and mortality remaining stable. Around one-quarter of patients died within 1 year, with men having more than twice the risk of 30-day and 1-year mortality than women. Patients who were older, frailer, resided in non-metropolitan regions, and who were admitted from a RACF were also at increased risk of mortality.

The 20% increase in absolute annual number of hip fractures presents a challenge for health policy and resource allocation, since hospitalization, radiological examination, treatment, and follow-up of hip fractures are highly resource-intensive [28]. The stable age- and sex-adjusted incidence rate ratios across the years suggest that the absolute increase was driven by Australia’s aging population [6]. The stable trend is in contrast to the decline in age-adjusted incidence reported using similar hospital admission data sources across Australia in earlier years from 2005 to 2015 [6]. This suggests that the effect of drivers for previous decline, such as increased screening and introduction of pharmacological treatment for osteoporosis [29-31], has plateaued. Additionally, the age-adjusted incidence rate ratios, comparing against the first year, peaked at earlier years in women while men peaked in the last year. Such differential trends in age-adjusted incidences between men and women were consistent with previous reports in Australia and may also explain the stable trend [6, 29-31]. However, as our population was large, the small statistically significant differences should be interpreted with caution. The increasing absolute numbers and the stabilized age- and sex-adjusted trend highlight the importance of continuing to monitor hip fracture incidence for appropriate resource allocation [8].

Hip fracture mortality in Victoria was comparable to the average hip fracture mortality rate (22.0% $\pm$ 7.2%) across 36 countries included in a recent systematic review [32]. The stable trend in Victoria was in contrast to decreasing mortality following hip fracture surgery in New South Wales from 2011 to 2018 [33]. Moreover, more than 80% of hospitals in New South Wales, in addition to the routinely collected administrative data, participated in patient-level audits that contributed to the Australian & New Zealand Hip Fracture Registry (ANZHFR) [34]. Increased participation in clinical audits has been shown to improve clinical practice [35]. In comparison, only 50% of Victorian hospitals participated in patient-level audits contributing to the Registry.

Patients with higher frailty scores had increased risk of 30-day and 1-year mortality after adjustment for age, sex, region, and type of residence. Patients with high and intermediate frailty risk had similar association with 30-day mortality, while patients with high frailty risk showed stronger association with 1-year than with 30-day mortality. Our results were consistent with pooled results of a systematic review on frailty and mortality in patients with hip fractures [13]. Most studies included in the systematic review used frailty assessments performed by clinicians. We demonstrated similar associations using the HFRS computed with routinely collected administrative data. This suggests the potential value of administrative data for targeting post-fracture care to those at highest risk of mortality.

Our results showed that admission from a RACF was associated with increased 30-day and 1-year mortality. The proportion of patients coded as being admitted from a RACF was lower than that in the ANZHFR [34]. It is possible that admission from a RACF was underestimated. However, the association with increased mortality was consistent with residents of RACFs having lower baseline health status. Although our analyses were adjusted for age and frailty status, the HFRS did not include parameters such as activities of daily living [23]. Patients discharged to RACFs are often excluded from studies of rehabilitation interventions [36]. Future research on hip fracture rehabilitation services specifically designed for different RACFs that provide different levels of care is warranted [37].

Patients from non-metropolitan regions had increased risk of 30-day and 1-year mortality compared to patients from metropolitan regions. This could partly reflect variation in access to specialized care, including timely access to hip fracture surgery and post-fracture rehabilitation [38, 39]. It may also reflect differences in health-seeking behaviors in rural and regional compared to metropolitan areas [40]. Lower socioeconomic status in non-metropolitan regions has also been associated with less intensive management of comorbidities [41]. However, no information on socioeconomic status independent of residence was available to delineate the effect.

Our results were congruent with previous studies that have reported higher risk of post-fracture mortality among men [42, 43]. This could be due to higher rate of pre-existing comorbidities and post-fracture infections, and lower use of pre- and post-fracture osteoporosis medications [42, 44]. Additional studies are needed to understand how the usage of pre-injury medications differed across sex in patients with hip fractures in Victoria and the impact on post-fracture outcomes and mortality.

### Strengths and limitations

Our study is the first in Victoria to investigate hip fracture incidence and mortality using real-world administrative data. Limitations common with other administrative data studies include possible under-reporting of hip fractures [19], differential recording of diagnoses and admission sources, incomplete medical records, wrong dates of admission, discharge, and deaths. Data quality were maximized with regular Australian Government data integrity audits [45] and the quality of ICD-10-AM coding in Victorian hospitals was previously validated [46]. We did not have information on time to surgery. However, majority of surgeries in Victoria are performed within the recommended timeframe [47]. Detailed information on ethnic background and socioeconomic status was not available. However, we included region of residence in our investigation to account for some of the social factors that may affect mortality following hip fracture [41]. We did not investigate the type of hip fractures. However, a previous Australian study found that the most common type of hip fractures was at the neck of the femur (56%) and pertrochanteric (or intertrochanteric) fractures (38%) [7]. We did not investigate individual comorbidities, which may be associated with higher risk of mortality following hip fracture [48, 49]. Future research on individual comorbidities is warranted. We instead used HFRS, a composite score from 109 diagnoses codes, to investigate burden of diseases on patients. HFRS has been validated in Australian administrative hospital data to assess mortality risk [50].

## Conclusion

The 20% increase in absolute annual number of hip fractures presents a challenge for health policy and planning. Men and those who are older, frailer, and reside in a RACF or non-metropolitan areas are at higher risk post-fracture mortality and may benefit from targeted interventions.

### Supplementary Information

Below is the link to the electronic supplementary material.

Supplementary file1 (DOCX 50.3 KB)

## References

[1] Harvey N, Dennison E, Cooper C (2010). Osteoporosis: impact on health and economics. Nat Rev Rheumatol.

[2] Gullberg B, Johnell O, Kanis JA (1997). World-wide projections for hip fracture. Osteoporos Int.

[3] Ballane G, Cauley JA, Luckey MM, Fuleihan GEH (2014). Secular trends in hip fractures worldwide: opposing trends East versus West. J Bone Miner Res.

[4] Wu AM, Bisignano C, James SL (2021). Global, regional, and national burden of bone fractures in 204 countries and territories, 1990–2019: a systematic analysis from the Global Burden of Disease Study 2019. Lancet Healthy Longev.

[5] Cheung C-L, Ang SB, Chadha M (2018). An updated hip fracture projection in Asia: the Asian Federation of Osteoporosis Societies study. Osteoporos Sarcopenia.

[6] Australian Governement Productivity Commission (2013) An ageing Australia: preparing for the future. Commission Research Paper, Canberra

[7] Australian Institute of Health Welfare (2018) Hip fracture incidence and hospitalisations in Australia 2015–16. AIHW, Canberra

[8] Cauley JA, Chalhoub D, Kassem AM, Fuleihan Gel H (2014). Geographic and ethnic disparities in osteoporotic fractures. Nat Rev Endocrinol.

[9] Tatangelo G, Watts J, Lim K (2019). The cost of osteoporosis, osteopenia, and associated fractures in Australia in 2017. J Bone Miner Res.

[10] Danford NC, Greisberg JK, Jobin CM, Rosenwasser MP, Walker MD (2021) Geriatric hip fractures: a practical approach. Springer Cham, Switzerland

[11] Araiza-Nava B, Méndez-Sánchez L, Clark P, Peralta-Pedrero ML, Javaid MK, Calo M, Martínez-Hernández BM, Guzmán-Jiménez F (2022). Short- and long-term prognostic factors associated with functional recovery in elderly patients with hip fracture: a systematic review. Osteoporos Int.

[12] Xue QL (2011). The frailty syndrome: definition and natural history. Clin Geriatr Med.

[13] Ma Y, Wang A, Lou Y, Peng D, Jiang Z, Xia T (2022). Effects of frailty on outcomes following surgery among patients with hip fractures: a systematic review and meta-analysis. Front Med (Lausanne).

[14] Brennan SL, Pasco JA, Urquhart DM, Oldenburg B, Hanna FS, Wluka AE (2010). The association between urban or rural locality and hip fracture in community-based adults: a systematic review. J Epidemiol Community Health.

[15] Chang W, Lv H, Feng C, Yuwen P, Wei N, Chen W, Zhang Y (2018). Preventable risk factors of mortality after hip fracture surgery: systematic review and meta-analysis. Int J Surg.

[16] Australian Bureau of Statistics (2019) Australian Demographic Statistics, Jun 2019. https://www.abs.gov.au/AUSSTATS/abs@.nsf/DetailsPage/3101.0Jun%202019. Accessed 1 April 2023

[17] Llopis-Cardona F, Armero C, Hurtado I, García-Sempere A, Peiró S, Rodríguez-Bernal CL, Sanfélix-Gimeno G (2022). Incidence of subsequent hip fracture and mortality in elderly patients. A multistate population-based cohort study in Eastern Spain. J Bone Miner Res.

[18] Walsh ME, Ferris H, Coughlan T, Hurson C, Ahern E, Sorensen J, Brent L (2021). Trends in hip fracture care in the Republic of Ireland from 2013 to 2018: results from the Irish Hip Fracture Database. Osteoporos Int.

[19] Thuy Trinh LT, Achat H, Loh SM, Pascoe R, Assareh H, Stubbs J, Guevarra V (2018). Validity of routinely collected data in identifying hip fractures at a major tertiary hospital in Australia. Health Inf Manag.

[20] Safe Care Victoria (2011) Victorian additions to the Australian Coding Standards effective 1 July 2011. https://www.bettersafercare.vic.gov.au/resources/clinical-coding-and-classifications/victorian-additions-to-the-australian-coding-standards-effective-1-july-2011. Accessed 1 December 2022

[21] Toson B, Harvey LA, Close JCT (2015). The ICD-10 Charlson Comorbidity Index predicted mortality but not resource utilization following hip fracture. J Clin Epidemiol.

[22] Quan H, Sundararajan V, Halfon P, Fong A, Burnand B, Luthi JC, Saunders LD, Beck CA, Feasby TE, Ghali WA (2005). Coding algorithms for defining comorbidities in ICD-9-CM and ICD-10 administrative data. Med Care.

[23] Gilbert T, Neuburger J, Kraindler J (2018). Development and validation of a Hospital Frailty Risk Score focusing on older people in acute care settings using electronic hospital records: an observational study. Lancet.

[24] Department of Health (2012) Victorian Admitted Episodes Dataset (VAED): accessible and restricted data fields. https://www.safercare.vic.gov.au/sites/default/files/2018-09/Victorian%20Admitted%20Episodes%20Dataset%20_VAED_%20Accessible%20%26%20Restricted%20Data%20Fields.pdf. Accessed 1 April 2023

[25] Australian Bureau of Statistics (2022) National, state and territory population: data downloads - time series spreadsheets (Population - Victoria). https://www.abs.gov.au/statistics/people/population/national-state-and-territory-population/latest-release#data-download. Accessed 1 December 2022

[26] Australian Bureau of Statistics (2013) Feature article: which population to use for age standardisation? https://www.abs.gov.au/ausstats/abs@.nsf/Lookup/3101.0Feature+Article1Mar%202013. Accessed 1 December 2022

[27] Robertson MC, Campbell AJ, Herbison P (2005). Statistical analysis of efficacy in falls prevention trials. J Gerontol A Biol Sci Med Sci.

[28] Jennifer Watts AJ, Abimanyi-Ochom J, Sanders KM (2013) Osteoporosis costing all Australian: a new burden of disease analysis - 2012 to 2022. Osteoporosis Australia, Melbourne

[29] Cassell E, Clapperton A (2013). A decreasing trend in fall-related hip fracture incidence in Victoria, Australia. Osteoporos Int.

[30] Crisp A, Dixon T, Jones G, Cumming RG, Laslett LL, Bhatia K, Webster A, Ebeling PR (2012). Declining incidence of osteoporotic hip fracture in Australia. Arch Osteoporos.

[31] Fisher AA, O'Brien ED, Davis MW (2009). Trends in hip fracture epidemiology in Australia: possible impact of bisphosphonates and hormone replacement therapy. Bone.

[32] Downey C, Kelly M, Quinlan JF (2019). Changing trends in the mortality rate at 1-year post hip fracture - a systematic review. World J Orthop.

[33] Harvey L, Harris IA, Mitchell RJ, Webster A, Cameron ID, Jorm L, Seymour H, Sarrami P, Close J (2022). Improved survival rates after hip fracture surgery in New South Wales, 2011–2018. Med J Aust.

[34] ANZHFR (2021) Annual Report of Hip Fracture Care 2021. Australian and New Zealand Hip Fracture Registry, Australia

[35] Hut-Mossel L, Ahaus K, Welker G, Gans R (2021). Understanding how and why audits work in improving the quality of hospital care: a systematic realist review. PLoS One.

[36] Sheehan KJ, Fitzgerald L, Hatherley S (2019). Inequity in rehabilitation interventions after hip fracture: a systematic review. Age Ageing.

[37] Royal Commission into Aged Care Quality and Safety (2021) Voume 1: summary and recommendations. Final report: care, dignity and respect commonwealth of Australia, Canberra

[38] Bohanna I, Harriss L, McDonald M (2021). A systematic review of disability, rehabilitation and lifestyle services in rural and remote Australia through the lens of the people-centred health care. Disabil Rehabil.

[39] Kosar CM, Loomer L, Ferdows NB, Trivedi AN, Panagiotou OA, Rahman M (2020). Assessment of rural-urban differences in postacute care utilization and outcomes among older US adults. JAMA Netw Open.

[40] Goodwin BC, March S, Ireland M, Williams FC, Manksi D, Ford M, Dunn J (2019). Geographic variation in compliance with Australian colorectal cancer screening programs: the role of attitudinal and cognitive traits. Rural Remote Health.

[41] Adair T, Lopez AD (2021). An egalitarian society? Widening inequalities in premature mortality from non-communicable diseases in Australia, 2006–16. Int J Epidemiol.

[42] Bajracharya R, Guralnik JM, Shardell MD, Rathbun AM, Yamashita T, Hochberg MC, Gruber-Baldini AL, Magaziner JS, Orwig DL (2022). Long-term sex differences in all-cause and infection-specific mortality post hip fracture. J Am Geriatr Soc.

[43] Kjærvik C, Gjertsen J-E, Stensland E, Saltyte-Benth J, Soereide O (2022) Modifiable and non-modifiable risk factors in hip fracture mortality in Norway, 2014 to 2018 : a linked multiregistry study. Bone Joint J 104-B:884–893 10.1302/0301-620X.104B7.BJJ-2021-1806.R1 PMC9251134 35775181

[44] Brozek W, Reichardt B, Zwerina J, Dimai HP, Klaushofer K, Zwettler E (2016). Antiresorptive therapy and risk of mortality and refracture in osteoporosis-related hip fracture: a nationwide study. Osteoporos Int.

[45] Victorian Agency for Health Information (2022) Health data integrity program. https://vahi.vic.gov.au/ourwork/health-data-integrity-program. Accessed 1 December 2022

[46] Henderson T, Shepheard J, Sundararajan V (2006). Quality of diagnosis and procedure coding in ICD-10 administrative data. Med Care.

[47] Harvey LA, Harris IA, Mitchell RJ, Webster A, Cameron ID, Jorm LR, Seymour H, Sarrami P, Close JCT (2021). Impact of pre-surgery hospital transfer on time to surgery and 30-day mortality for people with hip fractures. Med J Aust.

[48] McHugh MA, Wilson JL, Schaffer NE, Olsen EC, Perdue A, Ahn J, Hake ME (2023). Preoperative comorbidities associated with early mortality in hip fracture patients: a multicenter study. J Am Acad Orthop Surg.

[49] Atthakomol P, Manosroi W, Phinyo P, Pipanmekaporn T, Vaseenon T, Rojanasthien S (2020). Prognostic factors for all-cause mortality in Thai patients with fragility fracture of hip: comorbidities and laboratory evaluations. Medicina (Kaunas).

[50] Sharma Y, Horwood C, Hakendorf P, Shahi R, Thompson C (2022). External validation of the Hospital Frailty-Risk Score in predicting clinical outcomes in older heart-failure patients in Australia. J Clin Med.

