# Correction to: Hip fracture incidence and post-fracture mortality in Victoria, Australia: a state-wide cohort study

**DOI:** 10.1007/s11657-023-01286-y

**Published:** 2023-05-22

**Authors:** Miriam T. Y. Leung, Clara Marquina, Justin P. Turner, Jenni Ilomaki, Tim Tran, J. Simon Bell

**Affiliations:** 1 Centre for Medicine Use and Safety, Faculty of Pharmacy and Pharmaceutical Sciences, Monash University (Parkville Campus), 381 Royal Parade, Victoria 3052 Parkville, Melbourne, Australia; 2 Faculty of Pharmacy, University of Montreal, Québec, Canada; 3 Centre de Recherche, Institut Universitaire de Gériatrie de Montréal, Québec, Canada; 4 Faculty of Pharmacy, Laval University, Québec, Canada; 5 Department of Epidemiology and Preventive Medicine, Monash University, Melbourne, Australia; 6 Pharmacy Department, Austin Health, Melbourne, Australia; 7 Faculty of Health Sciences, University of Eastern Finland, Kuopio, Finland

**Correction to: Archives of Osteoporosis (2023) 18:56**

**https://doi.org/10.1007/s11657-023-01254-6**

The wrong Supplementary file was originally published with this article; it has now been replaced with the correct file.

### Supplementary information

Below is the link to the electronic supplementary material.

Supplementary file1 (DOCX 39 KB)

