# Supplementary material for: Hip fracture incidence and post-fracture mortality in Victoria, Australia: a state-wide cohort study
Source: Arch Osteoporos. 2023 Apr 29;18(1):56. doi: 10.1007/s11657-023-01254-6 (PMC10148778; doi:10.1007/s11657-023-01254-6)
Supplement: Supplementary file 1 — Supplementary file1 (DOCX 50.3 KB) [file 11657_2023_1254_MOESM1_ESM.docx]

**Supplemental Table 1.** ICD-10-AM codes for identification of comorbidities.

| Comorbidity | ICD-10-AM |
| --- | --- |
| Cardiovascular and cerebrovascular diseases | I00-I83, I85-I99, G45, Q20-Q26 |
| Chronic kidney disease | N00-N19, N25-N28, N39.1, N39.2, D59.3, B52.0, E85.3, Q60-Q63, T82.4, T86.1, Z49.0, Z94.0, Z99.2, U87.1 |
| Diabetes mellitus | E10, E11, E13, E14, O24 |
| Fall | W00-W19 |
| Osteoporosis | M80-M82 |

**Abbreviation: ICD-10-AM,** International Statistical Classification of Diseases and Related Health Problems, Tenth Revision, Australian Modification

**Supplemental Table 2.** Adjusted incidence rate ratios (IRR) of first incidence from 2012-13 to 2017-18 by sex.

| Parameter | IRR for overall incidence [95% CI]^a^ | IRR for overall incidence [95% CI]^b^ | IRR for incidence in females [95% CI]^b^ | IRR for incidence in males [95% CI]^b^ |
| --- | --- | --- | --- | --- |
| Year |  |  |  |  |
| 2012-13 | 1.00 (reference) | 1.00 (reference) | 1.00 (reference) | 1.00 (reference) |
| 2013-14 | 1.03 [0.95-1.12] | 1.02 [0.80-1.30] | 1.05 [1.00-1.10] | 1.04 [0.96-1.11] |
| 2014-15 | 1.07 [0.99-1.16] | 1.06 [0.84-1.35] | 1.12 [1.07-1.17] | 1.04 [0.97-1.12] |
| 2015-16 | 1.03 [0.95-1.12] | 1.03 [0.81-1.30] | 1.06 [1.01-1.11] | 1.01 [0.94-1.08] |
| 2016-17 | 1.02 [0.94-1.11] | 1.02 [0.80-1.29] | 1.03 [0.99-1.08] | 1.02 [0.95-1.10] |
| 2017-18 | 1.08 [1.00-1.17] | 1.08 [0.85-1.37] | 1.02 [0.97-1.07] | 1.10 [1.03-1.18] |
| Age |  |  |  |  |
| 50-64 | 1.00 (reference) | 1.00 (reference) | 1.00 (reference) | 1.00 (reference) |
| 65-74 | 3.83 [3.56-4.13] | 3.89 [3.20-4.74] | 4.48 [4.20-4.79] | 3.20 [2.96-3.47] |
| 75-84 | 15.5 [14.5-16.6] | 15.9 [13.1-19.3] | 18.6 [17.5-19.7] | 12.5 [11.7-13.5] |
| $\boldsymbol{\geq}$85 | 51.3 [47.9-55.0] | 51.8 [42.6-63.0] | 56.6 [53.5-59.9] | 45.7 [42.7-49.0] |
| Sex |  |  |  |  |
| Women | 1.00 (reference) |  |  |  |
| Men | 0.63 [0.60-0.66] |  |  |  |

**Abbreviations**: **IRR**, incidence rate ratio; **CI**, confidence interval. ^a^Adjusted for year, sex and age. ^b^Adjusted for year and age.

**Supplemental Table 3.** Adjusted mortality rate ratios (MRR) of 30-day and one-year mortality from 2012-13 to 2017-18 by sex.

| Parameter | 30-day mortality in females MRR [95% CI]^a^ | 30-day mortality in males MRR [95% CI]^a^ | One-year mortality in females MRR [95% CI]^a^ | One-year mortality in males MRR [95% CI]^a^ |
| --- | --- | --- | --- | --- |
| Year |  |  |  |  |
| 2012-13 | 1.00 (reference) | 1.00 (reference) | 1.00 (reference) | 1.00 (reference) |
| 2013-14 | 1.09 [0.85-1.40] | 0.96 [0.73-1.28] | 1.00 [0.80-1.24] | 1.02 [0.77-1.34] |
| 2014-15 | 1.14 [0.90-1.46] | 0.92 [0.70-1.22] | 1.08 [0.87-1.33] | 0.93 [0.71-1.22] |
| 2015-16 | 1.13 [0.88-1.44] | 0.94 [0.71-1.24] | 1.09 [0.88-1.36] | 1.01 [0.77-1.32] |
| 2016-17 | 1.05 [0.82-1.34] | 0.86 [0.65-1.14] | 1.02 [0.82-1.26] | 0.91 [0.69-1.18] |
| Age |  |  |  |  |
| 50-64 | 1.00 (reference) | 1.00 (reference) | 1.00 (reference) | 1.00 (reference) |
| 65-74 | 1.51 [0.86-2.75] | 1.37 [0.82-2.32] | 1.54 [1.07-2.21] | 2.71 [1.86-3.95] |
| 75-84 | 3.79 [2.32-6.53] | 4.25 [2.78-6.74] | 4.80 [3.48-6.64] | 11.6 [8.29-16.2] |
| $\boldsymbol{\geq}$85 | 9.49 [5.89-16.2] | 9.39 [6.22-14.5] | 17.6 [12.95-24.1] | 28.9 [20.9-40.1] |

**Abbreviations**: M**RR**, mortality rate ratio; **CI**, confidence interval. ^a^Ajusted for year and age .

**Supplemental Table 4.** Mortality rate ratios (MRR) of risk factors for 30-day mortality and 1-year mortality based on multivariate negative binomial regression, using diagnoses within 2 years prior discharge for hospital frailty risk score.

| Parameter | 30-day mortality MRR [95% CI]^a^ | 1-year mortality MRR [95% CI]^a^ |
| --- | --- | --- |
| Year |  |  |
| 2012-13 | 1.00 (reference) | 1.00 (reference) |
| 2013-14 | 1.04 [0.87-1.24] | 1.02 [0.86-1.20] |
| 2014-15 | 1.04 [0.88-1.24] | 1.01 [0.86-1.19] |
| 2015-16 | 1.01 [0.85-1.20] | 1.02 [0.86-1.20] |
| 2016-17 | 0.96 [0.81-1.15] | 0.95 [0.81-1.12] |
| Sex |  |  |
| Women | 1.00 (reference) | 1.00 (reference) |
| Men | 2.23 [1.99-2.50] | 2.74 [2.46-3.06] |
| Age |  |  |
| 30-64 | 1.00 (reference) | 1.00 (reference) |
| 65-74 | 1.33 [0.91-1.97] | 1.71 [1.31-2.23] |
| 75-84 | 3.37 [2.44-4.77] | 4.80 [3.79-6.09] |
| $\boldsymbol{\geq}$85 | 7.40 [5.39-10.4] | 15.6 [ 12.4-19.6] |
| Hospital frailty risk score^b^ |  |  |
| >0 and <5 | 1.00 (reference) | 1.00 (reference) |
| 5-15 | 1.60 [1.35-1.91] | 3.01 [2.57-3.52] |
| >15 | 1.68 [1.37-2.07] | 5.99 [4.98-7.21] |
| Type of residence |  |  |
| Home-dwelling and other^c^ | 1.00 (reference) | 1.00 (reference) |
| Residential aged care facilities | 2.46 [2.01-3.01] | 3.82 [3.09-4.73] |
| Region of residence^c^ |  |  |
| Metropolitan | 1.00 (reference) | 1.00 (reference) |
| Non-metropolitan | 1.17 [1.05-1.32] | 1.31 [1.18-1.47] |

**Abbreviations**: **MRR**, mortality rate ratio; **CI**, confidence interval. ^a^Adjusted for year, sex, age, HFRS, type and region of residence. ^b^Weighted score calculated using diagnoses within 2 years prior to and including index admission. ^c^Including admission from private residences, transition care programs, mental health accommodation and transfers from other health care organizations. ^d^Based on Department of Health Human Services Region classification.

**Supplemental Table 5.** Mortality rate ratios (MRR) of risk factors for 30-day mortality by sex, based on multivariate negative binomial regression.

| Parameter | 30-day mortality in women  MRR [95% CI]^a^ | 30-day mortality in men  MRR [95% CI]^a^ |
| --- | --- | --- |
| Year |  |  |
| 2012-13 | 1.00 (reference) | 1.00 (reference) |
| 2013-14 | 1.11 [0.87-1.42] | 0.97 [0.74-1.28] |
| 2014-15 | 1.16 [0.91-1.48] | 0.92 [0.70-1.21] |
| 2015-16 | 1.09 [0.86-1.39] | 0.91 [0.69-1.19] |
| 2016-17 | 1.07 [0.84-1.36] | 0.84 [0.64-1.10] |
| Age |  |  |
| 30-64 | 1.00 (reference) | 1.00 (reference) |
| 65-74 | 1.47 [0.84-2.68] | 1.27 [0.76-2.14] |
| 75-84 | 3.43 [2.10-5.93] | 3.56 [2.33-5.63] |
| $\boldsymbol{\geq}$85 | 8.57 [5.30-14.70] | 7.71 [5.11-12.10] |
| Hospital frailty risk score^b^ |  |  |
| 0 | 1.00 (reference) | 1.00 (reference) |
| >0 and <5 | 1.44 [1.18-1.73] | 1.36 [1.09-1.70] |
| 5-15 | 2.40 [2.00-2.88] | 2.45 [1.99-3.02] |
| >15 | 3.57 [2.41-5.30] | 3.61 [2.50-5.21] |
| Type of residence |  |  |
| Home-dwelling and other^c^ | 1.00 (reference) | 1.00 (reference) |
| Residential aged care facilities | 2.33 [1.75-3.11] | 2.26 [1.63-3.13] |
| Region of residence^c^ |  |  |
| Metropolitan | 1.00 (reference) | 1.00 (reference) |
| Non-metropolitan | 1.32 [1.12-1.55] | 1.11 [0.92-1.33] |

**Abbreviations:** **MRR**, mortality rate ratio; **CI**, confidence interval. ^a^Adjusted for year, age, HFRS, type and region of residence. ^b^Weighted score calculated using diagnoses within 2 years prior to admission. ^c^Including admission from private residences, transition care programs, mental health accommodation and transfers from other health care organizations. ^d^Based on Department of Health Human Services Region classification.

**Supplemental Table 6.** Mortality rate ratios (MRR) of risk factors for one-year mortality by sex, based on multivariate negative binomial regression.

| Parameter | One-year mortality in women  MRR [95% CI]^a^ | One-year mortality in men  MRR [95% CI]^a^ |
| --- | --- | --- |
| Year |  |  |
| 2012-13 | 1.00 (reference) | 1.00 (reference) |
| 2013-14 | 1.03 [0.83-1.27] | 1.08 [0.84-1.41] |
| 2014-15 | 1.08 [0.88-1.32] | 0.95 [0.74-1.23] |
| 2015-16 | 1.06 [0.87-1.30] | 0.99 [0.76-1.28] |
| 2016-17 | 1.04 [0.84-1.27] | 0.90 [0.70-1.16] |
| Age |  |  |
| 30-64 | 1.00 (reference) | 1.00 (reference) |
| 65-74 | 1.56 [1.10-2.23] | 2.22 [1.55-3.21] |
| 75-84 | 4.12 [3.01-5.66] | 7.69 [5.56-10.7] |
| $\boldsymbol{\geq}$85 | 15.8 [11.6-21.6] | 20.7 [ 15.1-28.6] |
| Hospital frailty risk score^b^ |  |  |
| 0 | 1.00 (reference) | 1.00 (reference) |
| >0 and <5 | 1.93 [1.65-2.26] | 1.92 [1.57-2.35] |
| 5-15 | 4.23 [3.61-4.96] | 4.64 [3.82-5.65] |
| >15 | 7.76 [5.50-11.1] | 8.46 [5.94-12.1] |
| Type of residence |  |  |
| Home-dwelling and other^c^ | 1.00 (reference) | 1.00 (reference) |
| Residential aged care facilities | 3.63 [2.80-4.73] | 3.19 [2.28-4.49] |
| Region of residence^c^ |  |  |
| Metropolitan | 1.00 (reference) | 1.00 (reference) |
| Non-metropolitan | 1.45 [1.27-1.67] | 1.22 [1.02-1.44] |

**Abbreviations:** **MRR**, mortality rate ratio; **CI**, confidence interval. Adjusted for year, age, HFRS, type and region of residence. ^b^Weighted score calculated using diagnoses within 2 years prior to admission. ^c^Including admission from private residences, transition care programs, mental health accommodation and transfers from other health care organizations. ^d^Based on Department of Health Human Services Region classification.
